# Cervical Osteomyelitis, Cardiac Arrest, and Lance-Adams Syndrome: A Case Report

**DOI:** 10.7759/cureus.23914

**Published:** 2022-04-07

**Authors:** Waiz Wasey, Caitlin Carter, Nav S Badesha, Maria Rossi, Malika Baig

**Affiliations:** 1 Family and Community Medicine, Southern Illinois University School of Medicine, Springfield, USA; 2 Psychiatry, Southern Illinois University School of Medicine, Springfield, USA; 3 Family Medicine, Southern Illinois University School of Medicine, Springfield, USA

**Keywords:** cardiac arrest, resuscitation, hypoxic injury, action myoclonus, myoclonus

## Abstract

Lance-Adams syndrome (LAS) is a rare complication of successful cardiopulmonary resuscitation, often accompanied by action myoclonus. Myoclonus may occur as generalized, focal, or multifocal movements and can include the face, trunk, and/or extremities. Only 100 cases of LAS have been reported worldwide. Here, we present the case of a 53-year-old female who had a cardiac arrest event after being admitted for posterior cervical wound dehiscence management following a posterior cervical fusion from C3-T1. The patient was successfully resuscitated but developed action myoclonus in all extremities shortly after. Anoxic brain injury and myoclonus led to debilitation and prolonged hospital stay. During her inpatient stay, she was treated with clonazepam, levetiracetam, and sodium valproate with mild improvement.

## Introduction

Lance-Adams syndrome (LAS) is a post-hypoxic syndrome characterized by myoclonus secondary to brain assault by cerebral hypoxia often following cardiopulmonary resuscitation [1]. With improved emergency services and resuscitation measures, this syndrome characterized by action myoclonus is receiving increasing attention; however, only a few 100 cases have been published to date.

LAS was first reported in 1963 by Lance and Adams [2] after observing muscle cramps in a patient who survived cardiac arrest. Brain imaging often shows no significant findings, just as in our case. Often uncontrollable, myoclonus can start anywhere from days to weeks following successful resuscitation [2]. In comatose patients, the onset might be within 12 hours [3]. The poorly understood etiology makes treatment a challenge in these patients.

Here, we present the case of a middle-aged adult who was admitted for treatment of cervical osteomyelitis and posterior cervical wound dehiscence after undergoing cervical fusion surgery. During the course of treatment, the patient developed LAS after an episode of cardiac arrest followed by successful resuscitation.

## Case presentation

A 53-year-old female was admitted to the hospital for management of posterior cervical wound dehiscence and spinal osteomyelitis, secondary to posterior cervical fusion that was performed a month ago. Infectious disease service started the patient on broad-spectrum antibiotics and plastic surgery planned for debridement and flap closure. She had no history of neurological deficits or seizures. She had a medical history of hypertension and diabetes and was on lisinopril, metformin, and carvedilol in the outpatient setting. Five minutes into receiving vancomycin, the patient had a cardiac arrest. She was resuscitated for 10 minutes prior to achieving the return of spontaneous circulation (ROSC). She was intubated and moved to the intensive care unit (ICU).

**Figure 1:**
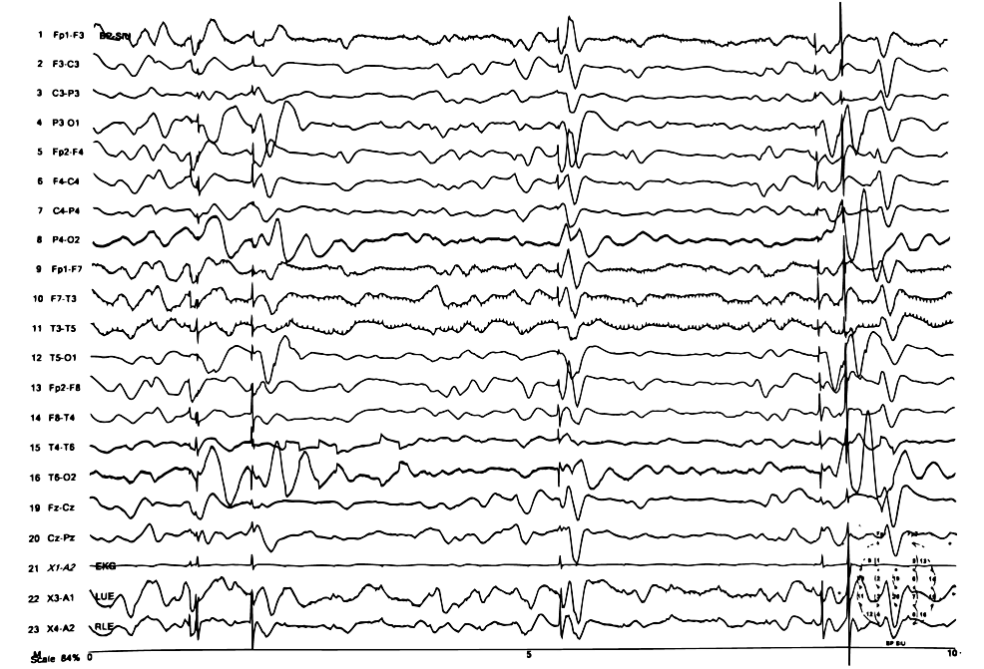
EEG showing generalized brain dysfunction. EEG: electroencephalogram

**Figure 2:**
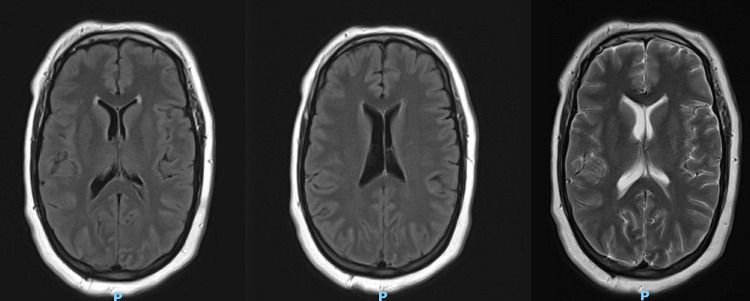
Normal brain MRI with and without contrast. MRI: magnetic resonance imaging

In the ICU, 8-12 hours post-resuscitation, the patient developed myoclonic jerks. They were noticeable mainly in the lower extremities with reduced sedation and tactile stimulus. An electroencephalogram (EEG) showed generalized brain dysfunction (Figure 1). An echocardiogram (Echo) showed good cardiac function with an ejection fraction of 55-60%. No deep vein thrombosis or pulmonary vein thrombosis was identified on imaging. Brain magnetic resonance imaging (MRI) (Figure 2) and computed tomography (CT) scan were unremarkable. After 72 hours in the ICU, neurology was consulted for persistent myoclonic jerks on reduced sedation. They did not rule out hypoxic injury and started the patient on levetiracetam. The neurological assessment showed intact brainstem reflexes.

The patient was started on clonazepam to control her myoclonic jerks. The frequency of these jerks reduced but persisted throughout the day. Soon after, the patient was successfully extubated. As she was unable to swallow without risk of aspiration, a percutaneous endoscopic gastrostomy tube was placed for nutrition. A physical therapy evaluation showed muscle strength of 3/5 in all extremities and an inability to perform small tasks due to persistent myoclonic jerking. The myoclonus was multifocal, action-induced, and associated with sudden lapses in muscle tone. Throughout the course, neurology adjusted medications by adding and increasing doses of valproic acid, levetiracetam, clonazepam, lorazepam, and baclofen. Only mild improvement was noted with drug therapy. Eventually, she was diagnosed with LAS, given no other organic causes for the myoclonus could be identified. Persistent myoclonus along with the inability of the patient to take care of herself led to the development of severe depression that further complicated her hospital stay with declining physical therapy progression. Psychiatry was also consulted to manage the underlying depression, and the patient was started on an antidepressant.

Due to the challenge in treating symptoms in this patient with mild improvement, she was discharged home with hospice. Neurology signed off after increasing clonazepam to a total daily dose of 18 mg.

## Discussion

Myoclonus is a neurological movement disorder that may be characterized as sudden, brief, shock-like jerky movements [4]. Traditionally, myoclonus is classified based on the source of origin in the neural pathway. Alternatively, it can be classified based on activity. Myoclonus occurring during an action is labeled action myoclonus [4].

LAS was first reported in 1963 by Lance and Adams who observed muscle cramps in patients who survived cardiac arrest. LAS is now defined as a rare neurological complication characterized by action myoclonus in successfully resuscitated patients due to brain injury from cerebral hypoxia [3]. Myoclonus may present within hours, days, or weeks of the event. Although the pathophysiology is poorly understood, the literature suggests a possible subcortical/cortical origin [5]. Loss of serotonin neurotransmitters within the inferior olive has been thought to be an important causal factor [6].

Brain imaging in these individuals may or may not reveal any findings. Most often, such as in our case, brain MRI is unremarkable. Other times, cortical ischemic changes might be noticed [5]. Further, EEG evaluations may be unremarkable. There have been reports of EEG showing a global or generalized slowing of brain activity without any seizure-like events. Our patient’s EEG showed generalized slowing of brain activity. Neuroimaging such as brain single-photon emission computed tomography or positron emission tomography may be of more diagnostic utility.

Treatment of LAS is limited with suboptimal success, mainly due to a lack of clear understanding of etiology. Clonazepam, sodium valproate, piracetam, and levetiracetam are recommended as first-line treatment options [7]. Our patient was treated with clonazepam, levetiracetam, and sodium valproate with no significant benefit. Perampanel, a recently introduced anti-epileptic drug, in low doses has shown improvements in the frequency and intensity of action myoclonus [8].

Because treatment is not 100% effective, patient care can be challenging. Our patient was depressed and debilitated as a result of profound action myoclonus in all of her extremities. Her hospital stay was prolonged due to depression, and she did not seem motivated to participate in physical therapy.

## Conclusions

LAS is a rare syndrome with very few reported cases, but because of more successful resuscitation measures, the incidence and awareness are increasing. Characterized by action myoclonus, LAS may cause debility and hinder the progress of recovery in patients. Poorly understood etiology poses a challenge in treating myoclonus. With a low success rate, anti-epileptic drugs have shown to be effective. This case report aims to highlight the importance of the detection of LAS and the challenge of treating it.
